# Exploring human-to-food contamination potential: Genome-based analysis of diversity, toxin repertoire, and antimicrobial resistance of methicillin-resistant *Staphylococcus aureus* in United Arab Emirates retail meat

**DOI:** 10.1016/j.onehlt.2025.101216

**Published:** 2025-09-19

**Authors:** Ihab Habib, Akela Ghazawi, Mohamed-Yousif Ibrahim Mohamed, Glindya Bhagya Lakshmi, Rania Nassar, Stefan Monecke, Ralf Ehricht, Danesh Moradigaravand, Dean Everett, Richard Goering, Mushtaq Khan, Abiola Senok

**Affiliations:** a Veterinary Public Health Research Laboratory, College of Agriculture and Veterinary Medicine, United Arab Emirates University, Al Ain, United Arab Emirates; b ASPIRE Research Institute for Food Security in the Drylands (ARIFSID), United Arab Emirates University, Al Ain, United Arab Emirates; c Department of Medical Microbiology and Immunology, College of Medicine and Health Sciences, United Arab Emirates University, Al Ain, United Arab Emirates; d College of Medicine, Mohammed Bin Rashid University of Medicine and Health Sciences, Dubai, United Arab Emirates; e Leibniz Institute of Photonic Technology (IPHT), Leibniz Center for Photonics in Infection Research (LPI), Jena, Germany; f InfectoGnostics Research Campus, Jena, Germany; g Institute of Physical Chemistry, Friedrich Schiller University, Jena, Germany; h Laboratory of Infectious Disease Epidemiology, Biological and Environmental Science and Engineering Division, King Abdullah University of Science and Technology, Thuwal, Saudi Arabia; i Department of Public Health and Epidemiology, College of Medicine and Health Sciences, Khalifa University, Abu Dhabi, United Arab Emirates; j Infection Research Unit, Khalifa University, Abu Dhabi, United Arab Emirates; k Department of Medical Microbiology and Immunology, Creighton University School of Medicine, USA; l Zayed Centre for Health Sciences, United Arab Emirates University, United Arab Emirates; m School of Dentistry, Cardiff University, Cardiff, United Kingdom

**Keywords:** Methicillin-resistant *Staphylococcus aureus*, Retail meat, Whole-genome sequencing, One health, United Arab Emirates

## Abstract

Methicillin-resistant *Staphylococcus aureus* (MRSA) has moved beyond healthcare settings and is increasingly documented in food, yet genomic data from the Arabian Peninsula are scarce. This study aimed to characterize the genomic diversity, antimicrobial-resistance (AMR), and virulence repertoire of MRSA in retail red meat sold in Dubai, United Arab Emirates (UAE), and to evaluate evidence for potential human-to-food transmission. In a cross-sectional survey (September 2024 – February 2025), 140 red-meat samples (beef, mutton, camel) were collected from supermarket chains. Fifty-one MRSA isolates were confirmed by multiplex PCR and phenotypic testing. Short-read whole-genome sequencing was followed by bioinformatic characterization of the isolates for clonal complex (CC) multilocus sequence typing (ST), *spa* typing, SCC*mec* assignment, AMR and virulence genes identification, plasmid-replicon detection, and core-genome phylogeny. Genotype, meat commodity, and products' origin associations were statistically assessed. Eight sequence types were detected; CC5-ST6 (27.5 %) and CC8-ST789 (23.5 %) predominated. SCC*mec* IV and V accounted for 82.3 % of genomes, and no livestock-associated CC398 was found. CC5/ST6-t304 and CC8-t091 comprised 47 % of isolates but showed no association with meat type (*p* = 0.451). Core-genome analysis split the collection into six clusters with shallow branch lengths, signaling recent clonal expansion across local and imported meat products. All genomes carried the methicillin resistance *mecA* gene plus a median of five additional resistance genes; 41 % carried multidrug-resistant determinants. Classical enterotoxin genes occurred in 80.4 % of isolates, and Panton–Valentine leukocidin genes in 27.5 %, mainly within CC8 and CC22. Distinct plasmid backbones—RepA_N–RepL–Rep_trans in CC5-ST6 and Inc18–Rep3 in CC8-ST789—were associated with lineage-specific AMR profiles. Retail red meat in the UAE is contaminated by clinically important, community-associated MRSA clones, likely introduced via human handling rather than animal reservoirs. Genomic surveillance at the human-food interface can guide regional One Health policies and help curb foodborne antimicrobial resistance dissemination.

## Highlights

• Red meat in the UAE harbors diverse MRSA clones commonly observed in clinical settings.
• The first genome-based survey of MRSA in UAE retail red meat reveals CC5 and CC8 dominance.
• Community-associated MRSA clones carrying SCC*mec* IV prevail across red meat types.
• Widespread multidrug resistance with frequent *mecA*, *blaZ*, *erm(C)*, and aminoglycoside genes.
• Distinct plasmid signatures in CC5 and CC8 underline lineage-specific AMR profiles.

## 1 Introduction

Methicillin-resistant *Staphylococcus aureus* (MRSA) represents a significant public health challenge worldwide, owing to its capacity to cause severe infections and its frequent multidrug-resistant phenotype [1]. Initially considered primarily as a hospital-associated pathogen, MRSA has increasingly emerged in community settings, raising concerns about its potential spread via novel transmission pathways, including food products [2,3]. The interface between humans, animals, and food commodities provides an intricate network facilitating the bidirectional transmission of MRSA, emphasizing the critical importance of a One Health approach to better understand and mitigate the risks associated with this pathogen [4].

Recent surveillance efforts have identified food products, particularly retail meat, as reservoirs for MRSA, suggesting that these products may serve as vectors for human exposure and subsequent colonization or infection [3,5]. A recent review revealed that MRSA was detected in 84.3 % of the studies (*n* = 165) conducted on retail foods, even though the actual sample-level prevalence often remained below 20 % [3]. This foodborne pathway presents a public health risk distinct from traditional healthcare or community exposures, warranting detailed genomic investigations to elucidate potential sources, transmission routes, and public health implications of MRSA contamination in the food supply chain.

The Arabian Peninsula, specifically the United Arab Emirates (UAE), represents a unique nexus for global trade in food commodities; approximately 90 % of the UAE's meat supply is imported from diverse geographic regions [6]. Such extensive international trade networks not only amplify the risk of cross-border transmission of antimicrobial-resistant (AMR) pathogens but also position the UAE as an ideal case study to explore foodborne AMR, including MRSA diversity, transmission dynamics, and human-to-food spillover potential within a globally interconnected food system.

Previous studies conducted in neighboring Gulf Cooperation Council (GCC) countries have identified clinically important MRSA lineages, notably Clonal Complex (CC) 5, in both healthcare settings and retail meat products, pointing to potential human-origin contamination of the food chain [7,8]. Detecting these clinically relevant strains in food commodities underscores the hypothesis of a spillover scenario whereby human-associated MRSA clones contaminate retail meat products through inadequate hygiene practices, infected food handlers, or cross-contamination during meat processing and handling [9]. To better understand and mitigate the risks associated with this pathogen, genomic characterization of MRSA isolated from retail meats can provide detailed insights into pathogen population structure, resistance and virulence gene profiles, as well as elucidate potential transmission pathways [10]. Whole-genome sequencing (WGS) offers unparalleled resolution for pathogen tracking and has been successfully deployed to investigate MRSA transmission dynamics across healthcare, community, and food environments in various global contexts [7,8].

This study provides the first genome-based characterization of MRSA in retail red meat from the UAE. Using WGS, we investigated the population structure, antimicrobial resistance determinants, toxin gene repertoire, and plasmid profiles of isolates recovered from different meat types presented at retail in Dubai. Our analysis further assessed the potential for human-to-food contamination, underscoring the public health risks associated with MRSA contamination in the food chain. Given the UAE's role as a major trade hub, these findings contribute not only to national food safety but also to broader global efforts in mitigating antimicrobial resistance in the food supply chain.

## 2 Materials and methods

### 2.1 Sample collection and identification of MRSA

A cross-sectional survey was conducted between September 2024 and February 2025, sampling different food commodities from supermarket outlets distributed across Dubai. The present article focuses on characterizing 51 MRSA isolates recovered from a total of 140 randomly sampled red-meat specimens: beef (*n* = 70), mutton (*n* = 50), and camel meat (*n* = 20) that were purchased as boneless cuts, bone-in pieces, or minced portions. Samples were collected from chilled supermarket displays, aseptically transferred into sterile containers, chilled (< 4 °C), and delivered to the laboratory within three hours for immediate microbiological processing. From each, a 25 g subsample was homogenized in 225 mL Mueller–Hinton broth containing 6.5 % NaCl and incubated (37 °C, 18–24 h) [11]. Ten-microliter loopfuls from enriched cultures were streaked on CHROMagar-MRSA (CHROMagar Microbiology, France) and re-incubated (37 °C, 18–24 h) [12]. Presumptive *S. aureus* colonies, i.e., colonies displaying rose/mauve colour, were sub-cultured on nutrient agar, and up to five distinct morphotypes per plate were screened. Species confirmation was performed by matrix-assisted laser desorption ionization–time of flight mass spectrometry (MALDI-TOF MS). (Autobio MS1000 (Autobio Diagnostics Co. Ltd., China)) [13].

Extracted genomic DNA (Wizard® kit, Promega, USA) from *S. aureus* confirmed colonies was subjected to multiplex PCR targeting 16S rRNA, *nuc,* and *mecA* to confirm methicillin resistance status [14]. Phenotypic resistance profiles were determined only for *mecA*-positive MRSA isolates using the VITEK-2 system (AST-P592 card; bioMérieux, France) [10]. The primary focus of this study is to provide an in-depth genomic characterization of the recovered isolates. Comprehensive prevalence data and phenotypic antimicrobial susceptibility testing (AST) results have been analyzed separately and are currently under peer review; therefore, they are not elaborated upon in the present manuscript.

### 2.2 WGS and bioinformatic analysis

For a total of 51 MRSA isolates from different types of red meat samples from UAE retailers, DNA libraries were prepared with the Nextera XT kit and sequenced on an Illumina NextSeq 2000 platform (2 × 150 bp) (Illumina, Inc., San Diego, CA, USA). Raw reads were processed through an in-house workflow [10]: (i) quality trimming with fastp v0.23.4, (ii) de novo assembly with SPAdes v3.15.5 and quality assessment via QUAST v5.2.0, (iii) multi-locus sequence typing (MLST) assignment (mlst v2.23) and *spa*-typing (spaTyper v1.0), (iv) SCC*mec* typing with SCC*mec*Finder v1.2, (v) antimicrobial resistance gene detection with AMRFinderPlus v3.12.1, (vi) toxin and virulence screening against the Virulence Factor Database (VFDB, accessed February 2025), (vii) plasmid replicon detection using PlasmidFinder v2.1, and (viii) phylogenetic tree analysis using the Solu platform v1.0.229 (accessed 15 February 2025), which incorporated core-genome alignment followed by maximum-likelihood phylogeny in IQ-TREE v2.2.2.6. Interactive visualization and metadata integration were performed in iTOL v6.9.0 [15,16]. Sequence data and accompanying metadata have been submitted to the National Center for Biotechnology Information (NCBI) under Bioproject: PRJNA1281856 (https://www.ncbi.nlm.nih.gov/bioproject/1281856).

### 2.3 Statistical analysis

Associations between clonal complexes, *spa* types, meat commodity, import status, and retail outlet were tested with Pearson's χ^2^ (or Fisher's exact test where appropriate) using R v4.3.2. Significance was set at *p* < 0.05 [17]. The diversity of AMR, virulence determinants, and plasmid types were visualized using Julius.ai, and pairwise distances within phylogenetic clusters were compared using the Kruskal–Wallis test [18].

### 2.4 Ethical approval

The study protocol was approved by the Emirate of Dubai Scientific Research Ethics Committee, Dubai Health Authority (DSREC-09/2023_10).

## 3 Results

### 3.1 Population structure of meat-borne MRSA isolates

**Fig. 1:**
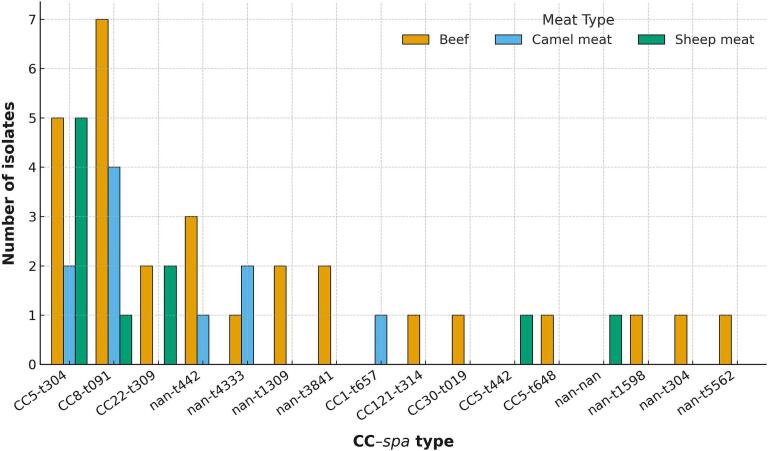
Distribution of paired clonal complex (CC)–*spa* type categories among 51 whole-genome sequenced methicillin-resistant *Staphylococcus aureus* (MRSA) isolates recovered from various red meat types sampled in Dubai, United Arab Emirates; nan = not assigned to a CC. (For interpretation of the references to colour in this figure legend, the reader is referred to the web version of this article.)

**Fig. 2:**
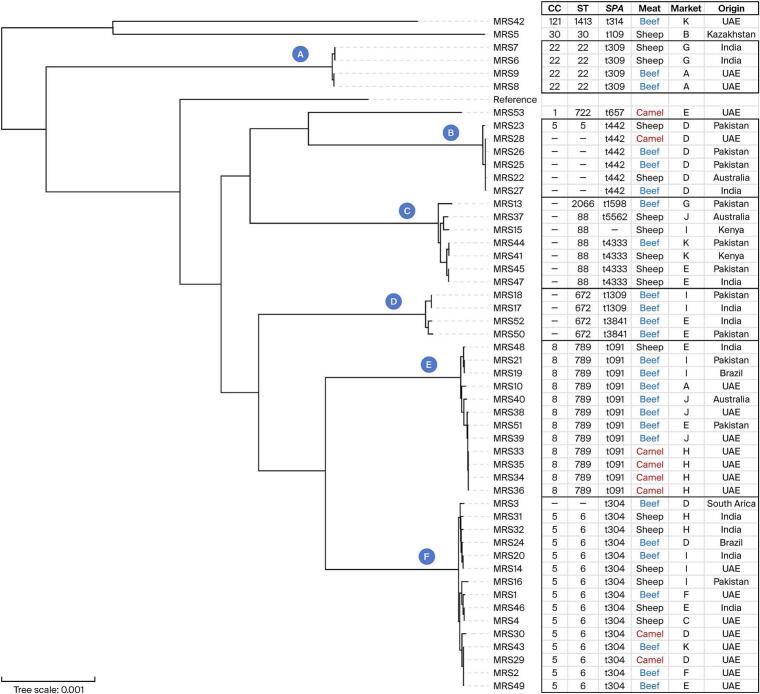
Maximum likelihood phylogenetic tree (midpoint rooted) of 51 whole-genome sequenced methicillin-resistant *Staphylococcus aureus* (MRSA) isolates obtained from different red meat types sampled in the Dubai, United Arab Emirates market. The tree was constructed using *Staphylococcus aureus* strain NCTC 8325 (assembly GCF_000013425.1) as the reference root. Clusters of closely related isolates (A to F) are indicated, with details on isolates clonal complex (CC), sequence type (ST), *spa*-type (*SPA*), along with data on meat type (MEAT), source market (MARKET), and country of origin (ORIGIN) shown on the right side of the tree. Isolates that do not belong to any CC, ST, *spa*-type it may be clearer to indicate this with a dash (“—”). (For interpretation of the references to colour in this figure legend, the reader is referred to the web version of this article.)

The isolates characterized in this study were sourced from beef (26/51, 51.0 %), followed by sheep meat (17/51, 33.3 %) and camel meat (8/51, 15.7 %) (Fig. 1). WGS of the 51 MRSA isolates obtained from retail red-meat products in Dubai revealed a markedly skewed sequence-type spectrum (Fig. 2). ST6 accounted for 27.5 % of the collection (14/51), followed by ST789 at 23.5 % (12/51). Three further lineages—ST88, ST672, and ST22—together contributed 27.4 %, while each of the remaining five STs was represented by a single genome (Fig. 2).

The distribution of *spa* types mirrored this pattern, with t304 (25.5 %) and t091 (23.5 %) jointly representing almost half of the isolates (Fig. 2). It was evident also that some isolates could not be characterized by *spa*-typing and/or MLST (Fig. 2). Clonal-complex assignment showed CC5 (29.4 %) and CC8 (23.5 %) as the major meat-associated lineages, whereas CC22, CC30, CC121 and CC1 together made up a minority (Fig. 2). Consistent with the predominance of community-acquired lineages, 62.7 % of the genomes carried SCC*mec* type IV and a further 19.6 % harbored type V, while 17.6 % were untypeable. The untypeable isolates may be attributed to factors such as suboptimal sequence quality or the presence of divergent/novel SCC*mec* elements not yet captured in available databases.

### 3.2 Paired CC–spa categories across meat commodities

When CC and *spa* data were combined, twenty CC–*spa* genotype categories were discerned (Fig. 1). Two categories—CC5/ST6-t304 and CC8-t091—dominated, each comprising 12 genomes (23.5 %). CC22-t309 (7.8 %), CC5-t648 (3.9 %), and a heterogeneous set of MRSA isolates that could not be assigned to known clonal complexes but carried *spa* types t442 or t4333 brought the cumulative total of the five most common categories to 78.4 % (Fig. 1). Assigning isolates against meat type showed that CC5/ST6-t304 was split almost evenly between beef and sheep meat, CC8-t091 was confined to beef and sheep meat, and CC22-t309 was distributed equally between beef and sheep meat (Fig. 1). In contrast, camel-meat genomes were confined mainly to unassigned CC–*spa* categories (Fig. 1&2). A three-way χ^2^ test yielded *p* = 0.451, indicating no significant association between genotype category and meat commodity (irrespective of the animal source).

### 3.3 Whole-genome phylogenetic relationships

The core-genome maximum-likelihood tree divided the 51 meat-borne MRSA genomes into six well-supported clusters, labelled A through F, each corresponding to a dominant sequence-type background (Fig. 2). Cluster F (ST6/CC5; *n* = 14) formed an intensely compact clade in which most pairwise branch lengths were < 0.001 substitutions per site. Although highly clonal, these isolates originated from beef, sheep and camel meat in roughly equal proportions and were split almost evenly between local UAE products (57 %) and imports, mainly from India (Fig. 2). Cluster E (ST789/CC8; *n* = 12) showed an analogous topography but differed in commodity composition, with one third of genomes recovered from camel meat and the remainder from beef and a single sheep meat sample; 58 % were domestic while the rest traced back to four producing countries (Fig. 2).

Clusters A and C were smaller yet equally heterogeneous. Cluster A (ST22/CC22; *n* = 4) contained genomes derived equally from beef and sheep meat, and from both local and imported sources. Cluster C (ST88; *n* = 6) was strikingly skewed toward sheep meat (83 %) and was exclusively imported from Pakistan, Kenya, India, and Australia. Cluster D comprised four ST672 genomes, all recovered from beef and imported from India or Pakistan (Fig. 2).

A χ^2^ test comparing cluster membership with meat commodity yielded no significant association (*p* = 0.071), confirming that meat animal sources do not determine the significant phylogenetic clustering. In contrast, cluster membership showed a modest but significant association with import status (*p* = 0.043), driven by clusters C and D, which were exclusively imported (Fig. 2). Overlaying retail-market codes onto the tree revealed substantial mixing: cluster F alone appeared in seven different retail outlets, and individual outlets often stocked isolates from several clusters simultaneously (Fig. 2). Thus, the phylogeny depicts a scenario in which a handful of successful MRSA lineages—principally ST6/CC5 and ST789/CC8—dominate the retail red-meat reservoir; yet do so against a background of considerable diversity in commodity, country of origin and point-of-sale.

### 3.4 Antimicrobial-resistance gene profile

**Fig. 3:**
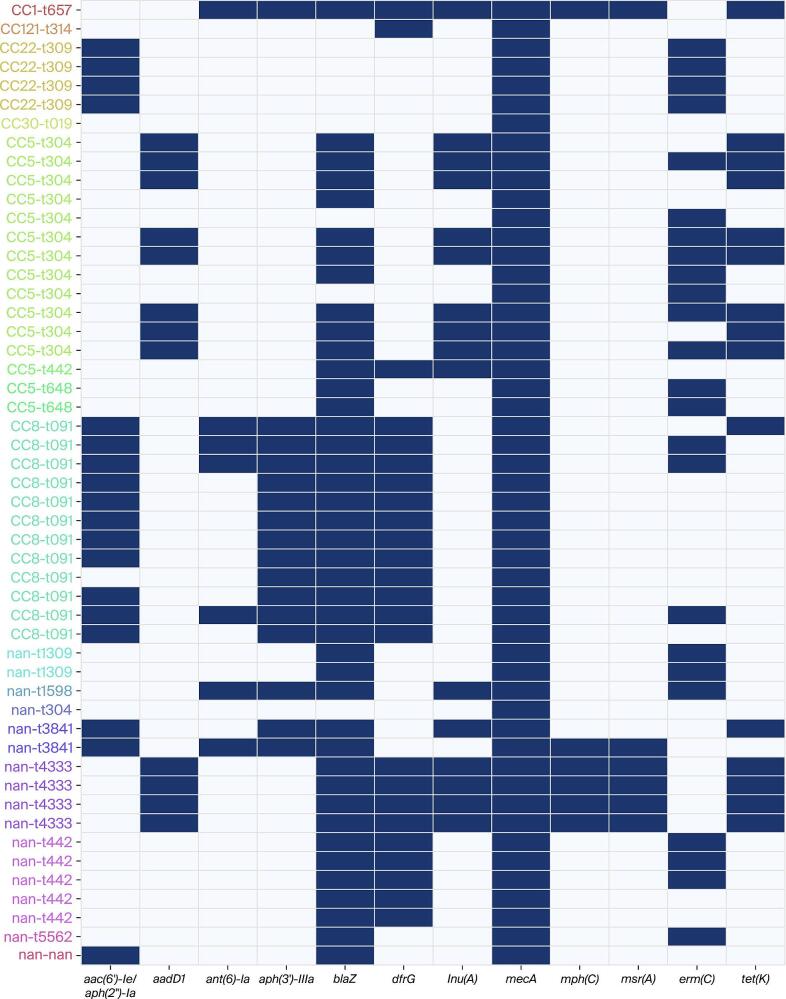
Antimicrobial resistance genes in relation to the paired clonal complex (CC)–*spa*-type categories (in various colors on y-axis) among 51 whole-genome sequenced methicillin-resistant *Staphylococcus aureus* (MRSA) isolates recovered from various red meat types sampled in Dubai, United Arab Emirates. Dark blue cells indicate the presence of a gene in the aligned isolates; nan = not assigned to a CC and/or *spa*-type. (For interpretation of the references to colour in this figure legend, the reader is referred to the web version of this article.)

As presented in Fig. 3, all genomes possessed *mecA*, confirming the methicillin-resistant phenotype. The number of additional resistance determinants per genome ranged from three to six. The β-lactamase gene *blaZ* occurred in 82.4 % of isolates, while *dfrG* (trimethoprim resistance) and *erm(C)* (macrolide-lincosamide–streptogramin phenotype) were each present in 47.1 %. Aminoglycoside resistance determinants were common, with *aac(6′)-Ie/aph(2″)-Ia* in 35.3 % and *aph(3′)-IIIa* in 31.4 % of the isolates. Tetracycline resistance driven by *tet(K)* was detected in 29.4 %, and Macrolide–Lincosamide–Streptogramin (MLS) efflux genes *mph(C)* and *msr(A)* appeared in 11.8 % of the genomes (Fig. 3). Lineage-specific patterns were evident: CC5-t304 genomes were enriched for *aac(6′)-Ie/aph(2″)-Ia* and *tet(K)*, whereas CC8-t091 genomes carried higher frequencies of *erm(C)* and *aph(3′)-IIIa* (Fig. 3). Forty-one per cent of isolates (21/51) possessed at least five distinct resistance genes, underscoring the widespread multidrug potential within the retail MRSA pool (Fig. 3).

### 3.5 Toxin gene repertoire

**Fig. 4:**
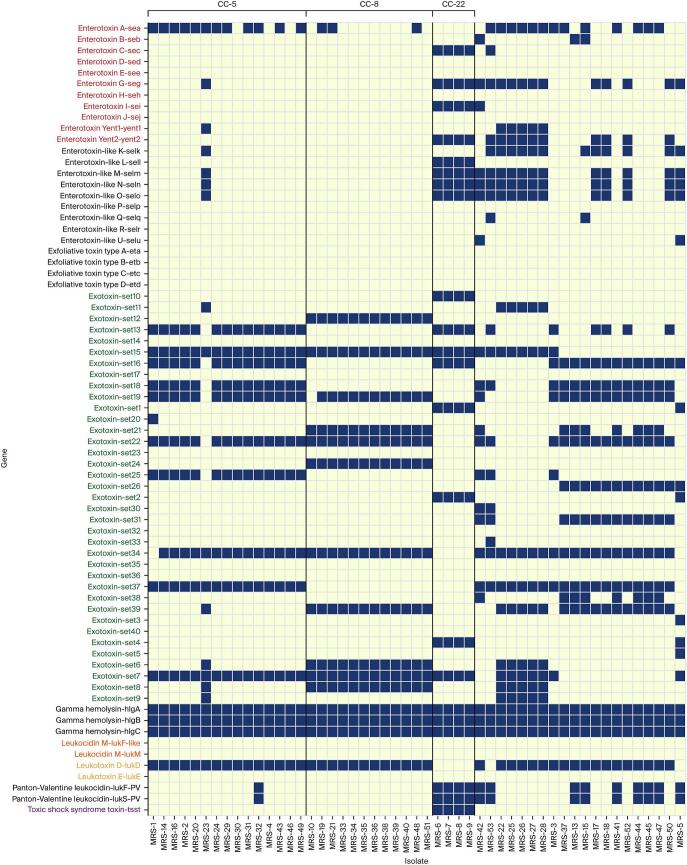
Toxin classes repertoire (in various colors on y-axis) across 51 whole-genome sequenced methicillin-resistant *Staphylococcus aureus* (MRSA) isolates recovered from various red meat types sampled in Dubai, United Arab Emirates. Dark blue cells indicate the presence of a gene in the aligned isolates. Isolates are grouped in relation to the three main CCs (CC-5, CC-8, and CC-22) as indicated on top part of the figure, visualizing presence (blue) or absence (yellow) of lineage-specific trends in virulence gene carriage. (For interpretation of the references to colour in this figure legend, the reader is referred to the web version of this article.)

Seventy-three toxin loci spanning sixteen functional classes were screened based on WGS analysis (Fig. 4). Alpha hemolysin (*hla*) and the γ-hemolysin operon were universally present, and δ-hemolysin (*hld*) appeared in 98 % of genomes, yet β-hemolysin (*hlb*) was entirely absent. Classical staphylococcal enterotoxin (*Se*) genes were widespread: *sea* (encodes staphylococcal enterotoxin A – a heat-stable superantigen causing food poisoning) was the most common (28/51, 54.9 %), followed by *seg* (33.3 %), while *seb* and *sec* were detected sporadically, and *sed* or *see* were not observed (Fig. 4). Overall, 80.4 % of genomes carried at least one classical or enterotoxin-like gene. Panton–Valentine leucocidin (PVL) genes (*lukF-PV / lukS-PV*; encode cytotoxins that destroy leukocytes and contribute to skin and soft tissue infections) were present in 27.5 % of isolates and concentrated within CC22-t309 and CC8-t091 lineages (Fig. 4).

The toxic-shock-syndrome toxin gene (*tst*) was restricted to four CC5 genomes (7.8 %). Analysis by meat type showed that enterotoxin genes were detected in 80.8 % of beef isolates, 88.2 % of sheep meat isolates, and only 37.5 % of camel-meat isolates. PVL prevalence was 47.1 % in mutton isolates but fell to 19.2 % and 12.5 % in beef and camel isolates, respectively, whereas *tst* occurred exclusively in beef and mutton genomes (Fig. 4).

### 3.6 Distribution of plasmid-replicon types

**Fig. 5:**
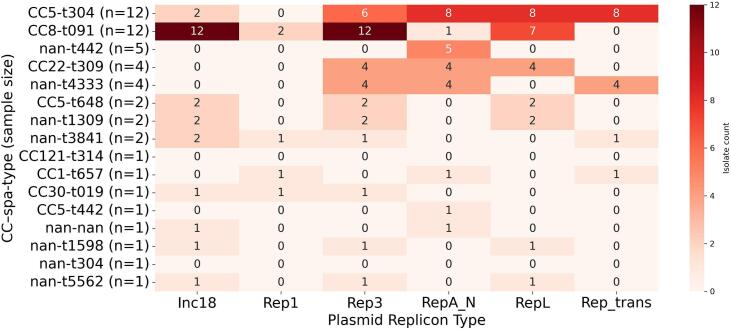
Lineage-specific distribution of plasmid-replicon types across CC–*spa*-type groups (nan = not assigned to a CC) across 51 whole-genome sequenced methicillin-resistant *Staphylococcus aureus* (MRSA) isolates recovered from various red meat types sampled in Dubai, United Arab Emirates; nan = not assigned to a CC and/or *spa*-type. (For interpretation of the references to colour in this figure legend, the reader is referred to the web version of this article.)

The two dominant groups, CC5-t304 and CC8-t091, comprised almost half of all isolates. However, they differed markedly in the plasmid signatures they carried (Fig. 5). Within CC5-t304 a characteristic triad of RepA_N, RepL and Rep_trans appeared in most isolates, frequently accompanied by Rep3 and only occasionally by Inc18 (Fig. 5). CC8-t091, on the other hand, was built around an Inc18–Rep3 core that was present in every member of the lineage; RepL was often, though not invariably, added to this backbone, whereas RepA_N was encountered only once and Rep_trans was absent.

The medium-sized clusters added further nuance (Fig. 5). All five members of the t442 group carried RepA_N and none carried any other replicon. CC22-t309 displayed a cohesive multi-replicon plasmid, combining Rep3, RepA_N, and RepL in every isolate, whereas t4333 shared the Rep3/RepA_N core but substituted Rep_trans for RepL, indicating divergence of plasmid scaffolds even among lineages of comparable size.

## 4 Discussion

The study on the Global burden of bacterial antimicrobial resistance (1990–2021) revealed that the global deaths caused by MRSA experienced the most significant increase, rising from 57,200 in 1990 to 130,000 in 2021—more than doubling over the period [1]. Hence, addressing MRSA requires a robust approach integrating systems thinking to curb its spread and mitigate its impact across sectors. This genome-based study is the first to chart MRSA in retail red meat from the UAE. The study fills a gap in Arabian-Peninsula food-safety surveillance by coupling WGS with metadata on commodity types, origins, and points of sale. The findings enrich the current Gulf Cooperation Council (GCC) baseline–wide benchmarking. They also firmly position the One Health discussion on AMR at the critical intersection between human health and the food supply. The relevance of this study extends well beyond national borders, as the UAE—a global market where 90 % of meat is imported [6]—serves as a key hub to study AMR within the context of international meat trade and its public health implications.

A striking feature of the dataset in this study is the dominance of two community-associated MRSA lineages, ST6/CC5 and ST789/CC8, which accounted for just over half of all characterized isolates from red meat. The prominence of CC5 mirrors recent findings from neighboring Saudi Arabia, where CC5-SCC*mec*Vc-t311 emerged as the leading clone in both meat and clinical isolates [7]. Notably, a recent study in the UAE pointed out that CC5 was the most dominant lineage in MRSA from clinical isolates, and concluded that CC5 MRSA is now endemic within the healthcare system in the UAE [8]. Our findings on the predominance of CC5 in the UAE retail meat mirrors its dominance in clinical settings [8], suggesting bidirectional flow between clinical, community, and food environments, raising the possibility that contaminated meat could act as a secondary colonization vehicle for such a significant clone [19,20]. It might also be hypothesized that the cassette–plasmid package that makes this clone (CC5) successful in hospitals also suits it for survival in a retail environment (e.g., food and surfaces) [21]. Additionally, the findings from this study on the carriage of staphylococcal enterotoxins by strains in this lineage raise a specific food-poisoning hazard, beyond harboring the *mecA* gene.

On the other hand, the presence of CC8 echoes the regional up-trend of USA300-like strains in hospitals and the community [22]. Described initially in North American community outbreaks, USA300 has achieved pandemic status, having been identified across all continents [22,23]. In the UAE, CC8 prevalence has shown an upward trajectory and is now identified as a common MRSA CC lineage circulating in human clinical settings [8,23]. It is worth noting that the livestock-associated lineage CC398 MRSA—widely reported in Europe and closely linked to intensive swine farming [5]—has not been detected in our setting, likely reflecting the absence of such agricultural practices (e.g., raising pigs) in the UAE. Our findings suggest that red-meat contamination in the UAE is driven by human-adapted clones rather than conventional livestock reservoirs. Thus, contamination of retail meat by colonized or infected handlers facilitates the introduction of prevalent MRSA clones into the food chain, thereby promoting subsequent community transmission of these clinically significant clones.

When multilocus-sequence-typing and *spa* data were merged, CC5/ST6-t304 and CC8-t091 emerged as the most frequent genotype combinations, accounting for almost half the isolates. Identical pairings dominate hospital collections in neighboring Saudi Arabia [7,24], indicating a regional clone pool transcending the food–clinical divide. Crucially, in our study, statistical testing showed no correlation between genotype and meat type, pointing instead to post-slaughter mixing within the retail chain [5]. Next to the statistical evidence, the analysis of genomes of the meat-borne MRSA in our study highlighted the widespread presence (62.7 %) of SCC*mec* IV, which is the archetype of Community-Associated (CA)-MRSA [25]. Its dominance suggests the meat became contaminated after slaughter, most plausibly (given the sizable proportion of imported meats) from colonized food-handlers or abattoir staff, rather than from livestock lines that more often carry SCC*mec* V (only found in 19.6 % of the isolates in this study) or IX (Livestock-Associated (LA)-MRSA) [25]. Investigations in Riyadh, Saudi Arabia, have implicated cutting boards, display trays, and glove reuse as critical contamination points [26]; our data echo that scenario and underscore the need for strengthening hygiene and regular verification of food-handler training.

Core-genome phylogenetics resolved six well-supported clusters, two of which (CC5/ST6 and CC8/ST789) displayed very shallow branch lengths (<0.001 substitutions per site), a star-like topology that betrays recent clonal expansion with scant time for neutral mutation accumulation [27]. Previous studies noted that CC5/ST6 is widely presented in humans in the GCC region [8,24]. On the other hand, regional studies point out that CC8 and CC22 almost certainly arrive via colonized humans [7,24]. CC5's enterotoxin gene set makes it the lineage most likely to cause classic staphylococcal food poisoning if temperature abuse occurs [23,28]. The mixed profile revealed in the current study implies that post-slaughter/post-processing contamination is more important than initial animal carriage. Additionally, CC22 detection flags the ability of a modern hospital clone to move through non-clinical environments, illustrating the porous boundary between healthcare and food sectors [8]. Routine retail meat MRSA surveillance using WGS should stratify by clonal complex, and shifts from CC5/ST6 dominance toward CC8/CC22 surges could be an early warning of hygiene failures in processing or retail.

The accessory resistome was equally cosmopolitan: *blaZ*, *erm(C),* and *dfrG* each approached the 50 % prevalence mark, while aminoglycoside-modifying enzymes and tetracycline efflux pumps were common. These multidrug patterns mirror UAE clinical panels [23] and align with meat surveys from Saudi Arabia [8] and India [29]. That similarity has therapeutic implications and potential consumer risks, where empirical treatment regimens for foodborne MRSA infection may need adjustment to the clones circulating.

Virulence profiling was no less worrisome. PVL genes appeared in 27 % of meat isolates, concentrated in CC22-t309 and CC8-t091. As PVL is ineffective against animal leukocytes, this strongly suggests that these strains are primarily of human origin and were secondarily transmitted to livestock, not the other way around [30]. Regional hospital series report PVL in 36 % and *tst1* in 12 % of clinical MRSA strains, most linked to skin and soft-tissue infections or necrotizing pneumonia cases [8,23]. CC22- Epidemic MRSA (EMRSA)-15 and PVL-positive CC8 have been reported in Gulf hospitals [30]; finding the same lineages in retail meat suggests local clones are recirculating through food-handler networks. Although our study concludes that the meat prevalence is lower, the overlap in clone background argues that retail products can serve as silent vectors. Rapid PVL diagnostics are therefore justified in hospitals and outbreak investigations along the food chain [30]. The confinement of *tst1* to CC22 mirrors Middle Eastern hospital data [31] and merits surveillance given its link to toxic shock syndrome.

Plasmid epidemiology added further insights. CC5-t304 harbored a RepA_N–RepL–Rep_trans scaffold frequently laden with enterotoxin and resistance genes, whereas CC8-t091 carried an Inc18–Rep3 backbone. The contrast between the two major lineages found in our isolates collection suggests that although both have expanded successfully, they have done so while carried by distinct plasmid architectures. Inc18 plasmids, noted for broad host range, have mediated glycopeptide-resistance transfer from *Enterococcus* to *S. aureus* in Asian poultry supply chains [32]. Conversely, the RepA_N/RepL/Rep_trans triad typical of CC5 underscores that lineage's global association with multi-replicon plasmids [33]. Such lineage-specific chassis help explain the AMR patterns observed and highlight retail meat as a reservoir where mobile elements with worldwide public-health significance converge. These findings highlight the dual forces of vertical inheritance within successful lineages and horizontal exchange that reshuffles replicon modules, ultimately shaping the plasmid landscape observed across the isolates characterized in this study.

Taken together, the absence of commodity association, the dominance of community-associated SCC*mec* types IV and V, the presence of virulence factors associated with human strains and the overlap with regional clinical datasets converge on a single narrative: retail red meat in the UAE is a *spillover* of human-origin MRSA into livestock (anthropozoonosis), most likely introduced by colonized food handlers or cross-contamination on shared equipment [34,35]. Strengthening food handlers' screening, enforcing glove changes between carcasses, and implementing routine environmental swabbing in high-throughput butcheries are interventions whose benefits are worth investigating in the context of the current study setting.

## 5 Conclusion

This genome-wide survey reveals that retail red meat in the UAE harbors a diverse MRSA clones commonly observed in clinical settings dominated by CC5/ST6 and CC8, enriched for multidrug resistance and high-impact virulence factors such as PVL. A predominance of SCC*mec* IV in MRSA from UAE red-meat points to community-associated, likely human-origin, contamination with clones that combine low fitness cost, high mobility, and often enhanced virulence [25]. The findings extend the geographic reach of foodborne MRSA surveillance and inform actionable hygiene verification in the food supply chain. Embedding whole-genome typing into regional food-safety programs, aligning analytical pipelines across human and veterinary laboratories, and enforcing auditable hygiene standards throughout the meat sector will be pivotal to curbing the silent circulation of clinically significant MRSA clones across the food-human interface.

## CRediT authorship contribution statement

**Ihab Habib:** Writing – review & editing, Writing – original draft, Project administration, Methodology, Investigation, Formal analysis, Conceptualization. **Akela Ghazawi:** Writing – review & editing, Validation, Software, Resources, Formal analysis. **Mohamed-Yousif Ibrahim Mohamed:** Writing – review & editing, Project administration, Investigation, Formal analysis. **Glindya Bhagya Lakshmi:** Writing – review & editing, Investigation, Formal analysis, Data curation. **Rania Nassar:** Writing – review & editing, Validation, Software, Resources. **Stefan Monecke:** Writing – review & editing, Visualization, Validation, Software, Resources. **Ralf Ehricht:** Writing – review & editing, Visualization, Validation, Software, Resources. **Danesh Moradigaravand:** Writing – review & editing, Visualization, Validation, Data curation. **Dean Everett:** Writing – review & editing, Visualization, Validation, Supervision, Resources. **Richard Goering:** Writing – review & editing, Validation, Supervision, Project administration, Methodology, Funding acquisition. **Mushtaq Khan:** Writing – review & editing, Supervision, Resources, Project administration, Funding acquisition. **Abiola Senok:** Writing – review & editing, Validation, Supervision, Project administration, Methodology, Funding acquisition, Conceptualization.

## Ethical approval

The study protocol was approved by the Dubai Scientific Research Ethics Committee of Dubai Health Authority - United Arab Emirates (Reference: DSREC-09/2023_10).

## Declaration of generative AI and AI-assisted technologies in the writing process

During the preparation of this work, the authors used OpenAI (2025). ChatGPT [Large language model]. https://chatgpt.com, and Grammarly (Grammarly Inc., 2025) to assist only in correcting sentence structure, grammar, and style. After using these tools, the authors reviewed and edited the proposed changes and take full responsibility for the content of the publication.

## Funding

This research was funded by the United Arab Emirates–United States of America National Institutes of Health (NIH) Collaborative Awards (Grant #: AJF-NIH-1-MBRU).

## Declaration of competing interest

The authors declare that they have no known competing financial interests or personal relationships that could have appeared to influence the work reported in this paper.

## Data Availability

Data will be made available on request.

## References

[1] GBD (2024). 2021 Antimicrobial Resistance Collaborators, Global burden of bacterial antimicrobial resistance 1990–2021: a systematic analysis with forecasts to 2050. Lancet.

[2] Vanderhaeghen W., Hermans K., Haesebrouck F., Butaye P. (2010). Methicillin-resistant *Staphylococcus aureus* in food production animals. Epidemiol. Infect..

[3] Khalifa H.O. (2024). Fire under the ashes: a descriptive review on the prevalence of methicillin-resistant *Staphylococcus aureus* in the food supply chain. J. Agric. Food Res..

[4] Algammal A.M. (2020). Methicillin-resistant *Staphylococcus aureus*: one-health perspective on epidemiology, virulence, antibiotic resistance and zoonotic impact. Infect. Drug Resist..

[5] González-Machado C., Alonso-Calleja C., Capita R. (2024). Prevalence and types of methicillin-resistant *Staphylococcus aureus* in meat and meat products: a review. Food Microbiol..

[6] Maqsood S. (2025). Are Emirati consumers in United Arab Emirates open to alternative proteins? Insights into attitudes and willingness to replace animal protein sources. Front. Sustain. Food Syst..

[7] Alkuraythi D.M. (2023). Clonal flux and spread of *Staphylococcus aureus* from meat and patients in Saudi Arabia. Microorganisms.

[8] Boucherabine S. (2025). Methicillin-resistant *Staphylococcus aureus*: the shifting landscape in the United Arab Emirates. Antibiotics.

[9] Lowder B.V. (2009). Recent human-to-poultry host jump, adaptation and pandemic spread of *Staphylococcus aureus*. Proc. Natl. Acad. Sci. U. S. A..

[10] Habib I. (2024). *Staphylococcus* spp. in salad vegetables: biodiversity, antimicrobial resistance and first methicillin-resistant strains in the UAE food supply. Foods.

[11] Larsen J. (2017). Evaluation of a culture-based method for detecting livestock-associated MRSA in Denmark and Norway. Euro Surveill..

[12] Nahimana I., Francioli P., Blanc D.S. (2006). Evaluation of chromogenic media and ORSAB for MRSA surveillance cultures. Clin. Microbiol. Infect..

[13] Park J.H. (2021). Comparison of autof MS1000 and Bruker Biotyper MALDI-TOF MS for routine microbial identification. Biomed. Res. Int..

[14] Aksakal A., Önalan Ş., Okalin Ş.Ş. (2022). Investigation of 16S rRNA, *mecA* and *nuc* genes in staphylococci by real-time PCR. Turk. J. Vet. Res..

[15] Saratto T. (2025). Solu: a cloud platform for real-time genomic pathogen surveillance. BMC Bioinform..

[16] Letunic I., Bork P. (2024). Interactive tree of life (iTOL) v6: recent updates to the phylogenetic tree display and annotation tool. Nucleic Acids Res..

[17] R Core Team (2023).

[18] Julius Technologies (2025). Julius.Ai: AI-Assisted Data Visualization and Analysis Platform, San Francisco, USA. https://www.julius.ai.

[19] Davis M.F. (2012). Household transmission of MRSA and other staphylococci. Lancet Infect. Dis..

[20] Shepheard M.A. (2013). Historical zoonoses and other changes in host tropism of *Staphylococcus aureus* revealed by phylogenetic analysis. PloS One.

[21] Jones M.J. (2015). Improving transformation of *Staphylococcus aureus* CC1, CC5 and CC8. PloS One.

[22] Tabatabaie Poya P. (2025). Hypervirulent CC8 *Staphylococcus aureus* in Tehran hospital wastewaters. Int. J. Microbiol..

[23] Senok A. (2020). Genotyping of methicillin-resistant *Staphylococcus aureus* from the United Arab Emirates. Sci. Rep..

[24] Alhejaili A.Y. (2025). MRSA in Saudi Arabia: genomic evidence of clonal expansion and plasmid-driven resistance. Front. Microbiol..

[25] Reichmann N.T., Pinho M.G. (2017). Role of SCC*mec* type in resistance to oxacillin-cefoxitin synergy in MRSA. Sci. Rep..

[26] Raji M.A. (2016). Genetic characterization of *Staphylococcus aureus* from retail meat in Riyadh. Front. Microbiol..

[27] Kennedy A.D. (2008). Epidemic community-associated MRSA: recent clonal expansion and diversification. Proc. Natl. Acad. Sci. U. S. A..

[28] Li Y. (2015). Enterotoxin genes and *spa* genotypes of MRSA from a Chinese tertiary hospital. J. Clin. Diagn. Res..

[29] Zehra A. (2019). MRSA in retail meat from Punjab, India: prevalence and molecular typing. J. Glob. Antimicrob. Resist..

[30] Senok A. (2021). Lateral-flow immunoassay for Panton-valentine leukocidin detection in UAE MRSA. Front. Cell. Infect. Microbiol..

[31] Boswihi S.S., Verghese T., Udo E.E. (2022). Diversity of clonal complex 22 MRSA isolates in Kuwait hospitals. Front. Microbiol..

[32] Kohler V., Vaishampayan A., Grohmann E. (2018). Broad-host-range Inc18 plasmids: occurrence, spread and transfer mechanisms. Plasmid.

[33] Al-Trad E.A. (2023). Plasmidomic landscape of clinical MRSA isolates from Malaysia. Antibiotics.

[34] Ferreira J.S. (2014). Food-handler-associated MRSA in public hospitals in Salvador, Brazil. Food Control.

[35] Aung K.T. (2017). Prevalence of methicillin-resistant *Staphylococcus aureus* in retail food in Singapore. Antimicrob. Resist. Infect. Control.

